# Self-Reported Nutrition Education Received by Australian Midwives before and after Registration

**DOI:** 10.1155/2017/5289592

**Published:** 2017-09-06

**Authors:** Jamila Arrish, Heather Yeatman, Moira Williamson

**Affiliations:** ^1^School of Health and Society, Faculty of Social Sciences, University of Wollongong, Northfields Avenue, Wollongong, NSW 2522, Australia; ^2^School of Nursing, Faculty of Science, Medicine & Health, University of Wollongong, Northfields Avenue, Wollongong, NSW 2522, Australia; ^3^School of Nursing and Midwifery, Higher Education Division, Central Queensland University, 90 Goodchap Street, Noosaville, QLD 4566, Australia

## Abstract

Educating midwives to provide nutrition advice is essential. Limited research focuses on midwives' nutrition education. This paper explores self-reported nutrition education received by Australian midwives before and after registration. It draws on quantitative and qualitative data from a larger online survey conducted with the members of the Australian College of Midwives (response rate = 6.9%, *n* = 329). Descriptive and content analyses were used. Of the midwives, 79.3% (*n* = 261) reported receiving some nutrition education during, before, and/or after registration. However, some described this coverage as limited. It lacked sufficient focus on topics such as weight management, nutrition assessment, and nutrition for vulnerable groups. Continuing education often occurred through personal initiatives, such as the midwife enrolling in external courses or exploring issues on the Internet and with colleagues. The majority of participants indicated a need for increased nutrition education (94.2%, *n* = 310) and guidelines tailored for them to provide nutrition advice (87.8%, *n* = 289). Australian midwives may not be receiving adequate nutrition education to provide nutrition advice. Inclusion of evidence-based nutrition components in midwifery education and regular updates for practising midwives focusing on challenging nutrition issues is required to ensure that they are supported in this important role.

## 1. Introduction

Accumulating research points to links between prenatal diet and maternal and foetal health. Observational studies have identified an association between healthy diet during pregnancy and reduced gestational weight gain and lower risk of pregnancy complications such as preterm birth, preeclampsia, and reduced foetal growth [1]. Pregnant women have increased requirements for some nutrients such as folic acid [2] and iodine [3] that might not be obtained from diet alone and supplementation is recommended. Other important aspects of maternal diet during pregnancy include appropriate weight gain and weight management, food safety, and management of pregnancy symptoms such as nausea and vomiting, constipation, and heartburn. The importance of nutrition during pregnancy and the importance of nutrition support as a component of antenatal care have recently been reinforced by the World Health Organization [4].

Pregnant women are considered more accepting of nutrition advice [5]. May et al. [6] found positive changes in the dietary behaviour of pregnant women who were provided nutrition advice by their health care professionals compared to those who were not. Yet the provision of nutrition advice by health professionals to pregnant women is limited, with lack of practitioner education being indicated as one of the obstacles to the provision of such advice [7]. Research has reported that nutrition education received by doctors is inadequate [8, 9]; however, midwives' nutrition education has received little attention.

Midwives are one of the primary care providers of prenatal care in Australia. Midwives have been reported as the most frequent source of health information for Australian pregnant women [10] and their role in the provision of nutrition advice is clearly defined in the International Confederation of Midwives Essential Competencies [11]. However, midwives seem to lack adequate nutrition knowledge and skills [12]. This may be attributed to inadequate education provided in midwifery courses [12]. Previous research in the United Kingdom and Australia indicated midwives' lack of education on some topics such as obesity and weight management [13–16] or healthy eating [13]. Prior Australian research also identified that health professionals (including midwives) need continuing professional education about nutrition during pregnancy and breastfeeding [17].

A recent Australian study investigating nutrition education within midwifery programmes using a mixed-methods approach incorporating online surveys and interviews with the course coordinators of the programmes identified some gaps [18]. Although all surveyed programmes included nutrition content within their curricula, topics taught varied, and the number of total hours was low. The education seemed to be medically oriented and lacked focus on developing nutrition assessment skills or providing practical nutrition training [18]. Additionally, a review investigating online opportunities for professional development in nutrition in Australia identified the scarcity of such opportunities and various gaps within those available [19].

The aim of this study was to explore Australian midwives' recollections of the nutrition education they received during basic education and following registration and their perspectives regarding their preparation to provide nutrition advice.

## 2. Methods

### 2.1. Design

The study used a cross-sectional descriptive design.

### 2.2. Participants and Distribution

The members of the Australian College of Midwives (ACM) were invited in 2012 (*n* = 4770) to participate in the study through an invitation published in the ACM newsletter and an email invitation sent through the ACM office. The invitations contained a hyperlink to the survey. Two email reminders were subsequently sent at one month intervals. The survey included an information sheet explaining the purpose of the study and a statement indicating that the participants provided their consent if they completed the survey. A power calculation was undertaken, providing an estimated minimum sample size of 356, based on a total population of 4770, margin error of 5, and a confidence level of 95%.

### 2.3. Survey Development

Previous literature [20–22] was drawn upon to develop the survey instrument. Five experts, including two dietitians, two academics (with expertise in public health nutrition and midwifery), and a statistician, reviewed the survey. The survey was circulated to five research colleagues and five practising midwives as a pilot. The survey was consequently modified prior to dissemination. Survey Monkey Software was used to create the final format online (Survey Monkey Inc., Palo Alto, California, USA, https://www.surveymonkey.com). The full survey examined midwives' provision of nutrition advice during pregnancy, their knowledge, attitudes, and confidence in this area [23]. The survey results indicated that 93% of the midwives provide nutrition advice to pregnant women but their nutrition knowledge and confidence in providing general and specific nutrition-related advice were inadequate. Therefore, more focus needs to be directed to nutrition education received by midwives [23].

### 2.4. Survey Items

This paper reports on the seven questions related to nutrition education received by midwives during midwifery education and/or during practice. The first two questions included open-ended components. Participants were asked if they received nutrition education and if yes, to report the number of hours, number of sessions provided, and where they were provided. Participants who answered “yes” to one or both of the questions also were directed to provide the details of this education (including information on who provided it and the nutrition topics covered) from a drop down menu. Midwives' views were sought on receiving more nutrition education and having guidelines tailored for them to provide nutrition advice, as well as the reasons for such views. Eight demographic questions were included (gender, age, midwifery education, years of experience, place of practice, principal state/territory of work, level of maternity services, and areas of maternity practice).

### 2.5. Analysis

The Statistical Package for the Social Sciences Software (SPSS) version 22 (Armonk, NY: IBM Corp.) was used to report the quantitative data. Descriptive data were presented using frequencies and percentages. Participants provided additional information via text boxes. This was analysed in a descriptive manner using content analysis [24].

### 2.6. Ethical Approval

The University of Wollongong Health and Medical Human Research Ethics Committee (HE12/009) granted ethical approval for the study and it was carried out in accordance with The Code of Ethics of the Declaration of Helsinki [25].

## 3. Results

There were 393 responses, 64 of which were excluded (61 for missing data, one a duplicate, one response from the pilot, and one electronic), resulting in 329 complete surveys for analysis (final response rate of 6.9%, 329 of 4770).  Table 1 shows the participants' demographics. The majority were female (98.8%, *n* = 325), more than 50 years of age (45.9%, *n* = 151), employed for over 10 years (69.6%, *n* = 229), and resident in New South Wales and Queensland (52.9%, *n* = 174), had hospital-based midwifery training (53.2%, *n* = 175), and were employed in public hospitals (86.6%, *n* = 285) generally in regional and tertiary referral hospitals (67.7%, *n* = 223) rotating through antenatal, birthing, and postnatal areas (41.3%, *n* = 136).

Of the 329 respondents, 79.3% (*n* = 261) reported that they had received nutrition information or education during their midwifery education and/or during practice.  Table 2 presents details of the nutrition education received during midwifery education and/or after registration. It was mainly provided by midwives (67.4%, *n* = 176), followed by dietitians/nutritionists (56%, *n* = 147). Instruction by other health professionals and organisations and self-directed learning (SDL) also made a contribution (24.5%, *n* = 64).

The most frequent nutrition topics covered were nutrition during pregnancy, for example, the role of folate, iodine, or calcium; alcohol and pregnancy; and the healthy range of weight gain required for pregnant women during different stages of pregnancy (78.9%, 77.0%, and 75.1%, resp.) (Table 2). Other nutrition topics included managing nausea, constipation, or heartburn; food safety and preparation (e.g., listeria); breastfeeding; and gestational diabetes management. Topics reported to a lesser extent were managing weight and reviewing diet for nutrition requirements of pregnancy (44.1% and 42.5%, resp.). The least covered topics were nutrition during pregnancy and different cultural groups and nutrition and teenage pregnancy (20.7% and 20.7%, resp.). General nutrition topics were also covered to a lesser extent, including the role of vitamins and minerals in the body, general food safety, and prevention of chronic illnesses such as cancer and heart disease. In addition to the provided list of topics, a few midwives added other topics (Table 2).

One hundred thirty-three respondents (40.4%) provided details of their nutrition education during midwifery education. Of these, 42.9% (*n* = 57) provided approximate number of hours and/or sessions taught. The number of lectures was generally small, ranging from 1 to 6 lectures, while numbers of hours ranged from 10 minutes to 10 hours. Very few midwives (*n* = 6) reported receiving extensive coverage of nutrition (e.g., 30 hours or more), and when they did so, it had usually been provided as a separate unit that lasted for a semester or a year. The nutrition education was offered in a range of educational, clinical, and community settings.

Thirty-four (10.3%) midwives indicated that the nutrition education provided was limited, more focused on the basics or foods to avoid, and mostly integrated with other topics rather than being presented separately. According to one respondent, such education provided only a* very small amount of information about foods that pregnant women should avoid. (Participant 278)*

Details of their nutrition education during practice were provided by 149 respondents. Of these, 58 respondents reported the duration of education sessions provided after registration. Sessions ranged from 20 minutes or an hour for individual lectures/sessions to 120 hours for separate units or courses.

Self-directed learning (SDL) was reported by 62 midwives, sometimes as their main or only avenue to obtain nutrition education. Self-directed learning avenues included attending conferences/workshops; undertaking nutrition courses; reading scientific journals, books, and pamphlets; and communicating with other health professionals.

Some midwives reported receiving in-service nutrition education (*n* = 22), while others reported accessing such education at their own expense.

None offered. Have accessed 2 day study days at own cost. (Participant 282)

In response to the question on whether their practice would benefit from receiving more nutrition information or education, 94.2% (*n* = 310) of the midwives responded “yes,” 1.22% (*n* = 4) responded “no,” and 4.6% (*n* = 15) “have not thought about it.” Of the 329 midwives, 40.1% (*n* = 132) provided further details. Many of the midwives' written responses are related to professional commitment to their role. Many of the responses (59.1%,* n* = 78) indicated that more nutrition education would help improve their knowledge or keep it up to date, increase their confidence, and help them provide evidence-based, consistent advice.

Would feel more confident and knowledgeable in offering appropriate info[information] and advice re [on] diet and nutrition. (Participant 153)

A smaller group of midwives (*n* = 16) included comments that reflected a broad perspective of the importance of nutrition support to the health of the mother, baby, and the whole family.

Diet is key to good health and if we teach the mother how to eat right then we impact the whole family. We also reduce the complications of pregnancy if we get good diet and weight management. (Participant 287)

Some midwives (*n* = 10) expressed a desire for greater nutrition knowledge to help them provide accurate information in regard to more specific pregnancy-related issues, such as the prevention and management of obesity and diabetes, as well as allergies, and nutritional challenges associated with the culturally diverse women presenting.

We are seeing more obese women and it is difficult to give them advice on weight management during pregnancy. (Participant 115)

Ten midwives specifically expressed their belief that it was part of their professional role to provide nutrition advice to pregnant women.

Midwives are often the first contact a mother has with a health professional during her pregnancy and should be given every opportunity to expand knowledge and give the best advice to mothers and their families. (Participant 208)

Structural constraints also prompted midwives desire to seek greater nutrition education (*n* = 4); For example, their role was considered particularly important due to limited availability of dietitians' services.

Currently unable to refer women to[the] dietitian, [due to] (funding)… therefore midwives, especially those involved in continuity of care models are at the coal face to make changes given they have the right information and tools to do so. (Participant 107)

A few midwives (*n* = 4) believed they do not receive enough nutrition education despite their being the health professionals with the most contact with pregnant women.

Very little education or resources are directed towards midwives in this area and we are the health professionals who spend most time with the women. (Participant 311)

Some midwives (*n* = 10) either were opposed to receiving more information or had not thought about it or wanted more information but had reservations. Three thought that dietitians are better positioned to provide specialised advice and they should be seen by all pregnant women. Others (*n* = 4) thought that even if education was provided, it would need regular updates and might not be possible for every issue, specific enough, or conveyed to women due to time constraints. One midwife exhibited a lack of confidence in current dietary guidelines.

Yes I would like to do more study re [on] nutrition but I am not confident that our current dietary guidelines are leading our society to greater health and so I would not be keen to study “mainstream” dietary information. I believe though that good nutrition is the basis for good health and so is an essential aspect of pregnancy care. (Participant 232)

Some midwives (*n* = 14) indicated a range of topics they would like to have covered in ongoing education, including obesity, gestational diabetes, nutrients, diets for women from different cultural backgrounds or women living in remote areas, special diets, weight loss for postpartum women, and exercise. Three types of education were suggested by six midwives: hard copy information and references; guidelines in an easy access format; and regular updates such as in-service and professional development sessions.

The majority of the respondents (87.8%, *n* = 289) thought that Australian midwives would benefit from guidelines specifically tailored for them to provide pregnant women with nutrition advice; 3% (*n* = 10) disagreed, while 9.1% (*n* = 30) had not thought about it. When asked to explain their answers, 36.2% (*n* = 119) responded. Their explanations for why nutrition guidelines could be beneficial included that the guidelines would encourage midwives to provide more evidence-based, consistent advice and reduce conflicting information for both midwives and women (*n* = 52). It would also overcome the lack of clinical guidelines specific to Australia, unlike those for smoking and diabetes (*n* = 9). Given the importance of nutrition and midwives' role in nutrition advice (*n* = 15), clinical guidelines would overcome midwives' lack of nutrition knowledge, the current failure to uniformly provide adequate nutrition education in midwifery education and practice, and limited access to dietitians (*n* = 10).

Things work so much better when we eliminate some of the shades of grey…a consistent guideline would be terrific in many areas of maternity care, not just nutrition advice. (Participant 48)

A small number (*n* = 19) were in favour of guidelines tailored to their requirements but they had some reservations, while 18 midwives were not in favour of guidelines tailored to midwives' needs or had not thought about it. Reservations about such guidelines included the need for such guidelines to be evidence-based, user-friendly, and women-centred (and in one case have a naturopathic holistic approach), involve midwives in their development, and be regularly updated. One midwife commented that there are already available guidelines while another thought guidelines may restrict advice given.

Guidelines can be useful. However, yet another “tick box” would be annoying. Guidelines can potentially lead to prohibitive practice. (Participant 30)

Further reasons given against guidelines included that midwives need to collaborate with other health professionals (e.g., dietitians); and learning should be encouraged rather than merely relying on guidelines.

My concern would be a one size fits all doesn't work for everyone, what is out there tends to be very middle-class/income, Anglo-Saxon, older/motivated women centric. Teenagers will tell you they choose the healthy options at McDonald's (and mean it!). (Participant 91)

## 4. Discussion

This study explored Australian midwives' own accounts of the nutrition education they had received. The majority reported receiving some nutrition education during midwifery education and/or after registration; however, this education lacked focus on contemporary nutrition issues and was generally described as limited. The provision of nutrition education to the midwives was reported to be mainly provided by midwives with further involvement of professionals with relevant expertise, such as dietitians and to a lesser extent by obstetricians or other doctors. Some midwives reported attempting to address their lack of formal nutrition education through self-directed learning. The majority indicated a need for increased nutrition education and guidelines tailored for them to provide nutrition advice.

Midwives generally commented that the coverage of nutrition during midwifery education was limited, integrated within other topics, and focused on the basics. The reported number of lectures and/or hours was small. Few midwives reported having a separate unit covering nutrition during pregnancy. Recent research [18] surveyed Australian accredited midwifery programmes and found that the number of hours of nutrition education in half of the programmes was only 5 to >10 hours, with only two programmes having a separate nutrition unit. There was also a lack of approaches to develop nutrition assessment skills or have practical nutrition training [18].

Limited nutrition education in midwifery programmes raises the need for those providing preregistration education to review their coverage of nutrition. Midwives' nutrition knowledge has been significantly linked to nutrition education received during their initial education [23]. However, it is unrealistic to assume that preregistration nutrition content should suffice for all future practice, as professional knowledge is dynamic, evolving in line with the evidence base. Thus the role of preregistration nutrition education in implementing necessary knowledge and skills that can be improved during practice cannot be ignored.

Several challenges have been identified in the literature that impact effective integration of nutrition education within health professionals' education including obtaining time within the curricula to integrate nutrition; focusing on teaching the role of nutrients in metabolic pathways rather than focusing on practical food-based knowledge; lack of emphasis on nutrition; lack of involving nutrition experts; and a lack of resources [26]. A competency-based nutrition education addressing clinical and public health nutrition and interprofessional collaboration have been suggested as strategies to tackle these challenges [27].

In response to the lack of subsequent formal opportunities for nutrition education during practice, many of the midwives reported that they undertook self-directed learning or independent learning. Such learning [28] assumes that adult learners are mature, independent, self-directed, responsible, and individual, and their learning is connected to their social roles and previous experiences, so they need approaches where they are partners rather than passive receivers [29]. The overwhelming availability of new content (such as new areas of nutrition information) and competency-based education have increased the interest in SDL [29]. Although SDL is considered as the most suitable model for health professionals to keep their knowledge up to date, there are mixed results about the effectiveness of SDL models compared to traditional learning models [30]. It was found that it is more effective in improving knowledge than attitudes and skills [30]. It is also more suitable to advanced learners [29]. There are also no consistent means to determine the learner readiness to SDL and the nature of the content most appropriate to SDL [29].

The majority of the midwives in this study were mature in age and this might be the reason for referring to SDL as a way of obtaining nutrition education. However, the suitability of SDL in nutrition education remains unknown for younger midwives specifically and for midwives in general. Further research in this regard is needed. From our study self-directed learning seemed to involve some issues such as the outlay of personal funds (not all midwives may be in a position to do so) and the variable quality of the nutrition education received. Midwives would benefit from being offered affordable and easily accessible nutrition education from trusted midwifery organisations and their workplaces. Access to information from dietitians as the experts in the field would also benefit midwives.

Other midwives were the main providers of nutrition education to the participants. However, it was identified that midwives may themselves receive limited education in nutrition. The provision of nutrition education by professionals with relevant expertise, such as dietitians, was mentioned by around 60% of the midwives. This is not unexpected, as dietitians were found to be rarely involved in teaching nutrition content within Australian midwifery programmes [18]. Other research has suggested that collaboration between dietitians and midwifery academics in terms of nutrition education for midwives is feasible and could help improve midwives' nutrition knowledge [31].

Variable nutrition coverage within midwifery education and/or after registration may result in variable quality of midwifery practice in this important area. Primarily it focused on basic and theoretical topics. Complex topics and those incorporating practical or management skills (such as weight management and reviewing diet for nutrition requirements of pregnancy) were reported less frequently. Similar results have been reported previously [21, 32]. According to the ICM Essential Competencies, midwives should have the knowledge/skills to assess women nutritionally [11]. The ability of midwives to manage weight is essential given the high rates of overweight and obesity among childbearing women [33]. Midwives in this study listed the increasing rates of obesity as a reason for the need for greater nutrition information and specific guidelines. A previous study has found that Australian midwives using evidence-based guidelines to manage obesity were more likely to report adequate education and greater confidence in counselling [34].

Of concern was the identification of the least covered nutrition topics, particularly nutrition for different cultural groups, and nutrition and teenage pregnancy. Pregnant women from different cultural backgrounds and pregnant teenagers are nutritionally vulnerable groups [2]. The results of this study are consistent with previous international research that found that midwives lacked sufficient knowledge and skills to counsel pregnant women from different cultures [35]. Midwives specifically pointed out that they would benefit from greater knowledge/clinical guidelines and in-service education on diets of women from culturally diverse backgrounds that would help them in these circumstances.

It was not possible to distinguish which phase, either during midwifery education or practice, the midwives were referring to in their answers to the questions regarding the providers of nutrition education and the topics covered. Fifty-seven percent of the midwives were aged 41 and older; thus they may have received their midwifery education some time ago and 50% of the midwives reported that they had gained their education through the now abandoned hospital-based training rather than through university education. However, the pattern of topics reported as included within their received nutrition education was similar to topics reported to be covered in contemporary Australian midwifery programmes [18]. This may imply little change in the pattern of nutrition topics covered within midwifery education. It may not reflect the changes in nutrition knowledge and advances in the understanding of the importance of nutrition during pregnancy or the changes in midwifery practice, such as increasing numbers of women with obesity or diabetes, or the greater diversity of vulnerable women.

Midwives in this study believed in the importance of nutrition during pregnancy and their role in providing advice and therefore their need for more nutrition education and specific guidelines. The realisation by the midwives of the need to provide evidence-based, consistent advice is understandable. There is currently an overwhelming amount of nutrition information available in the media, particularly on the Internet, with many confusing messages that are not scientifically proven. Nutrition science continues to develop and new information is constantly emerging. This presents challenges for busy midwives. Formal updates from official organisations such as the ACM and their workplaces would ensure that midwives are up to date with the latest evidence-based advances in nutrition. Prior research has also shown that midwives' knowledge and confidence could be improved through compact training [22, 36]. Unfortunately, there is lack of the availability or provision of such intensive training in Australia [19].

Strategies for providing continuing education (e.g., online nutrition education) have some advantages, such as reaching a wide audience with high-quality content; being an efficient, relatively low-cost method of delivery; providing convenient, self-paced study; and being effective at building capacity. However, it requires funds to develop and sustain [26]. Unless there are initiatives from the government and the professional bodies to provide such training, continuing nutrition education for health professionals will remain a challenge.

At the time of administrating the survey, the only available dietary guidelines were the Australian Dietary Guidelines (2003, revised in 2013) which were general guidelines and did not include advice specific to particular health professionals, such as midwives (who are not extensively trained in nutrition), on how to provide nutrition advice to pregnant women. In 2012 and 2014, Australia released its first National Antenatal Clinical Guidelines [37, 38]. A small number of the midwives expressed reservations regarding guidelines tailored to midwives' needs and one midwife exhibited a lack of confidence in current dietary guidelines, which may reflect a tension between her personal views and her professional role. Future research could explore the views of midwives on the National Antenatal Clinical Guidelines; the support midwives should receive to incorporate the guidelines into midwifery practice; and the extent to which they are consistent with their expectations of their professional roles.

### 4.1. Limitations and Strengths

While the response rate was low (6.9% of the members of ACM), it was similar to a previous study conducted with this cohort [34]. Online surveys tend to have lower response rates compared to mailed surveys [39]. The majority of the midwives were of mature age. Therefore, many were not able to provide the details of the nutrition education they received during their midwifery education several decades earlier. This may be due to the use of open-ended questions to obtain such data. The authors chose to use open-ended questions to give the participants the freedom of reporting their own experiences instead of providing limited certain answers. The use of both quantitative and qualitative data is strength of this study. Quantitative data revealed that about 80% of the midwives received some nutrition education during midwifery education and/or practice but the qualitative data highlighted the shortcomings of this education. The varying responses to the various questions eliciting midwives' perceptions and needs did not permit exploration of associations of such responses with demographics or work characteristics of the participants.

### 4.2. Practical Implications

National general recommendations can be made to include nutrition in a systematic way in midwifery programmes through the Australian Nursing and Midwifery Accreditation Council. Midwives need continuous support from official and trusted organisations, such as the ACM and their workplaces, to provide them with regular evidence-based nutrition updates, especially in terms of contemporary issues such as obesity. These recommendations can be applicable internationally through similar official midwifery organisations.

### 4.3. Future Research

Younger midwives were underrepresented in this study; future research should focus on investigating newly graduated midwives' perceptions of nutrition education received during midwifery education and learning objectives regarding nutrition education within midwifery programmes to explore if skills to deal with challenging issues such as obesity are included in the curricula. Future studies also might explore the actual nutrition education being provided in midwifery programmes and how to make changes to curricula to facilitate the incorporation of nutrition education and the range of information that women seek from midwives, to ensure that the nutrition education within programmes is relevant.

## 5. Conclusion

Australian midwives may not be adequately prepared to provide nutrition advice, a role that is increasingly important due to greater number of pregnant women with nutrition-related issues. Limited coverage of nutrition within midwifery or continuing education was reported, with personal initiatives undertaken to address nutrition information gaps. A more systematic approach aimed at ensuring that all midwives have the basic nutrition knowledge and skills they require to provide nutrition advice to pregnant women, as outlined in the new Australian Antenatal Clinical Guidelines, is required.

## Figures and Tables

**Table 1:** Characteristics of the respondents.

Characteristics	Number of responses (*n*^*∗*a^)	Percentage (%)
Gender		
Female	325	98.8
Male	4	1.2
Age		
21–30 years	28	8.5
31–40 years	55	16.7
41–50 years	95	28.9
Older than 50 years	151	45.9
Education		
Bachelor degree of midwifery	74	22.5
Hospital-based training midwifery	175	53.2
Initial midwifery postgraduate degree	80	24.3
Years of experience		
Less than 2 years	22	6.7
2–5 years	30	9.1
6–10 years	48	14.6
More than 10 years	229	69.6
Principal state or territory of work		
New South Wales (NSW)	96	29.2
Queensland (QLD)	78	23.7
South Australia (SA)	30	9.1
Tasmania (TAS)	8	2.4
Victoria (VIC)	58	17.6
Western Australia (WA)	40	12.2
Australian Capital Territory (ACT)	8	2.4
Northern Territory (NT)	11	3.3
Principal place of practice		
Public hospital	285	86.6
Private hospital	16	4.9
Independent midwifery practice	28	8.5
Level of maternity services		
Community	53	16.1
Rural hospital	53	16.1
Regional hospital	113	34.3
Tertiary referral	110	33.4
Area of midwifery practice^b^		
Antenatal care	96	29.2
Birthing (labour) suite	54	16.4
Postnatal	89	27.1
Rotation through all the above areas	136	41.3
Group practice (case load or team midwifery)	67	20.4
Independent midwifery practice	28	8.5

^a^Total number = 329. ^b^Multiple responses allowed. ^*∗*^The table is reused with permission from Elsevier.

**Table 2:** Nutrition information/education details.

Nutrition education details	*n* ^a^	%
The providers of nutrition education^b^		
Midwives	176	67.4
Dietitians/nutritionists	147	56.3
Obstetricians or other doctors	36	13.8
Other^c^	64	24.5
I do not know	25	9.6
Nutrition topics covered in the education^b^		
Topics of nutrition during pregnancy		
Nutrition during pregnancy, for example, the role of folate, iodine, or calcium	206	78.9
Alcohol and pregnancy	201	77.0
The healthy range of weight gain required for pregnant women during different stages of pregnancy	196	75.1
Nutrition-related issues such as managing nausea, constipation, or heartburn	178	68.2
Food safety and preparation during pregnancy (e.g., listeria)	174	66.7
Nutrition for breastfeeding	173	66.3
Nutrition management of gestational diabetes	164	62.8
Managing weight during pregnancy	115	44.1
Reviewing diet for nutrition requirements of pregnancy	111	42.5
Nutrition during pregnancy and different cultural groups	54	20.7
Nutrition and teenage pregnancy	54	20.7
Topics of general nutrition		
General nutrient information, e.g. the role of vitamins and minerals in the body	124	47.5
General food safety	95	36.4
General nutrition, for example, prevention of chronic illnesses such as cancer and heart diseases	46	17.6
Other^d^	10	3.8

^a^ *n *= 261 (only midwives who received nutrition information/education during midwifery education or after registration answered this section). ^b^Multiple responses allowed. ^c^“Other” included self-directed learning (through internet, media, reading books, journals, and attending conferences), complementary therapists (such as naturopaths and homeopaths), chiropractors, diabetes educators, midwives (colleagues, lecturers, and presenters in conferences or online) drug companies representatives, drug and alcohol staff and nurses, nutrition experts, kinesiologists, International Board Certified Lactation Consultant (IBCLC) course educators, governmental organisations, health promotion officers, and interaction with pregnant women. ^d^“Other” included nutrition for newborn and infants, nutrition during labour and after birth (especially for women from different cultural backgrounds such as Asian, Muslim, and African women), nutrition for vegetarians and vegans, nutrition for fertility, nutrition for alleviating symptoms such as thrush, eczema, and allergies, nutrition for preconception, the impact of maternal nutrition on child health, and the effects of socioeconomic factors on nutrition status, supplements, and organic food.
